# Hybrid threshold adaptable quantum secret sharing scheme with reverse Huffman-Fibonacci-tree coding

**DOI:** 10.1038/srep31350

**Published:** 2016-08-12

**Authors:** Hong Lai, Jun Zhang, Ming-Xing Luo, Lei Pan, Josef Pieprzyk, Fuyuan Xiao, Mehmet A. Orgun

**Affiliations:** 1 School of Computer and Information Science, Southwest University, Chongqing 400715, China; 2 School of Information Technology, Deakin University, Geelong, VIC, 3220, Australia; 3 School of Information Science and Technology, Southwest Jiaotong University, Chengdu 610031, China; 4 School of EE&CS, Queensland University of Technology, Brisbane, Australia; 5 Institute of Computer Science, Polish Academy of Sciences, Warsaw, Poland; 6 Department of Computing, Macquarie University, Sydney, NSW 2109, Australia; 7 Faculty of Information Technology, Macau University of Science and Technology, Avenida Wai Long, Taipa, 999078, Macau

## Abstract

With prevalent attacks in communication, sharing a secret between communicating parties is an ongoing challenge. Moreover, it is important to integrate quantum solutions with classical secret sharing schemes with low computational cost for the real world use. This paper proposes a novel hybrid threshold adaptable quantum secret sharing scheme, using an *m*-bonacci orbital angular momentum (OAM) pump, Lagrange interpolation polynomials, and reverse Huffman-Fibonacci-tree coding. To be exact, we employ entangled states prepared by *m*-bonacci sequences to detect eavesdropping. Meanwhile, we encode *m*-bonacci sequences in Lagrange interpolation polynomials to generate the shares of a secret with reverse Huffman-Fibonacci-tree coding. The advantages of the proposed scheme is that it can detect eavesdropping without joint quantum operations, and permits secret sharing for an arbitrary but no less than threshold-value number of classical participants with much lower bandwidth. Also, in comparison with existing quantum secret sharing schemes, it still works when there are dynamic changes, such as the unavailability of some quantum channel, the arrival of new participants and the departure of participants. Finally, we provide security analysis of the new hybrid quantum secret sharing scheme and discuss its useful features for modern applications.

Secret sharing is an important and powerful tool for protecting confidentiality and integrity of sensitive information, such as missile launch codes, bank account information, medical information and encryption keys. Secret sharing can be categorized into two broad classes: classical and quantum. Secret sharing was invented in its classical form simultaneously by Shamir^1^ and Blakley^2^. The Shamir secret sharing splits a secret into multiple shares in such a way that a large enough collection of shares can be used to reconstruct the secret. The minimum number of shares that enables the reconstruction is called the threshold or in general the access structure. However, if the number of shares is smaller than the threshold, then they provide no information about the secret. In other words, when a fewer than the threshold number of shares are compromised, the secret cannot be revealed. Later many other secret sharing schemes^3^,^4^,^5^,^6^,^7^ have been proposed to improve the traditional ones. The obvious weakness of classical secret sharing is that an adversary can duplicate shares without being detected. As a result, eavesdropping attacks could happen in the reconstruction phase when the participants send their shares to a combiner who computes the secret.

To address the eavesdropping problem, Hillery *et al.*^8^ extended classical secret sharing (CSS) to a (*m*, *n*)-threshold quantum secret sharing (QSS), which is the generation of quantum key distribution (QKD)^9^,^10^,^11^. In their scheme, GHZ states are used to transmit the shares securely in the presence of eavesdroppers, like the method used in ref. 12. The security of their scheme is guaranteed by the quantum no-cloning theorem^13^. Following Hillery *et al.*’s work, many quantum secret sharing schemes^14^,^15^,^16^,^17^,^18^,^19^,^20^,^21^,^22^,^23^,^24^,^25^,^26^,^27^ have been proposed with rigorous security proofs as well as properties that make them suitable for many applications. Though quantum secret sharing can detect eavesdropping, Źukowski *et al.*^23^ argue that QSS is different from CSS. The main difference is that in QSS, the choice of parameters *m* and *n* is restricted while in CSS, the parameters can be arbitrarily selected as long as *n* ≥ *m*. In particular, in a (*m*, *n*)-threshold QSS, the parameters *m*, *n* must satisfy the condition, 2*m* − 1 > *n*, which is imposed by the quantum no-cloning theorem^13^. However, in practice, security policies and the adversary structure demand the parameters *m* and *n* to be flexible and scalable. Therefore, it is very challenging to develop a new (*m*, *n*)-threshold QSS scheme, whose parameters *m* and *n* are not restricted.

In this paper, we propose a hybrid quantum secret sharing scheme to address this challenge. The new scheme is free from any restrictions on the parameters *m* and *n* and therefore it is suitable for many real-world applications. We employ entangled states prepared by *m*-bonacci sequences to detect eavesdropping, which can be done by any subset of participants that contains at least 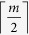 (ceiling 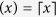 is the smallest integer greater than or equal to *x*) members. That is to say, not all participants are required to reach a consensus in order to reveal eavesdropping. We use *m*-bonacci sequences encoded in Lagrange interpolation polynomials to generate the secret, with no restrictions imposed on the parameters *m*, *n*. Given that *m*-bonacci numbers can be represented by Fibonacci numbers, we use the structure of the Huffman-Fibonacci tree with the greedy algorithm to encode *m*-bonacci sequences, i.e., the higher the frequency at which a Fibonacci number appears in *m*-bonacci sequences, the longer the block of binary codes. Therefore, our scheme can greatly improve the coding capacity, thus reducing the use of entangled photons, which are expensive and difficult to prepare. In real-world applications, some changes may occur when a new participant joins or alternatively, an existing participant leaves or there is a sudden disruption of some quantum channels. The new scheme has the capability to deal with such changes since the *m*-bonacci-number coding can be easily modified to reflect changes in secret sharing.

## Results

**Figure 1:**
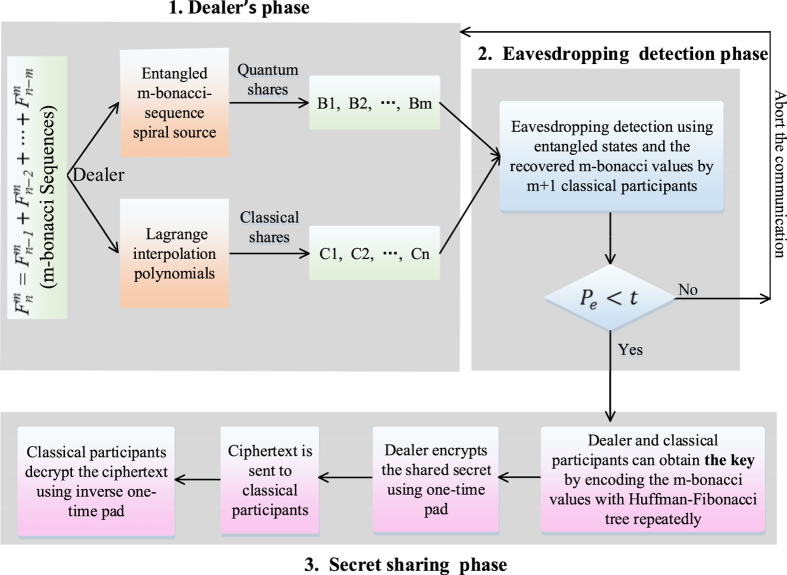
The sketch for the hybrid threshold adaptable QSS scheme which consists of three parts. 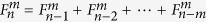
 represents *m*-bonacci sequences, *B*_1_, *B*_2_, ···, *B*_*m*_ denote *m* quantum shares, and *C*_1_, *C*_2_, ···, *C*_*n*_ denote *n* classical shares, *P*_*e*_ denotes the error rate and *t* the preset threshold value for *P*_*e*_.

In this section, we first describe a new hybrid quantum secret sharing based on *m*-bonacci sequences, as shown in Fig. 1. The scheme consists of two components: quantum and classical. The classical component allows to establish an infinite random sequence in a way of quantum encoding, which is shared by classical participants. The classical shares of the random sequence allow any *m* + 1 participants to recover the sequence. The one-time-pad encryption is done by a collection of *m* + 1 classical participants. The decryption can be done by any other collection of *m* + 1 classical participants. There are three phases in the proposed scheme:

- share generation and distribution – the dealer phase
- eavesdropping detection phase
- secret reconstruction phase

Then its security is analyzed and compared with other related QKD protocols.

### New hybrid threshold quantum secret sharing scheme

First, we introduce the generalized Fibonacci sequence^28^, that is so-called *m*-bonacci sequence which is used in our later scheme. The *m*-bonacci sequence of order *m* ≥ 2 denoted by 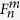 (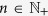) is defined by the following recurrence^28^,^29^:


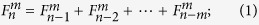


with the first *m* − 2 initial terms set to 0 and the (*m* − 1)th initial term set to 1. In particular, the 2-bonacci sequence is the usual Fibonacci sequence^30^; 3-bonacci sequence is usually called the Tribonacci sequence^28^. In Table 1, we list the first ten *m*-bonacci numbers when *m* = 2, 3, 4, 5, 6.

Then, we present the entities used in our secret sharing, which are as follows:

- a dealer,
- *m* (which is the same as *m* in *m*-bonacci numbers) participants who hold quantum shares (quantum participants),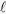 participants who hold classical shares (classical participants) and
- Adversaries.

**Dealer** is a party who is trusted by all quantum and classical participants. It is responsible for the initialization of the secret sharing. It generates shares and distributes them to all the participants. It is assumed that after finishing its tasks, the dealer “forgets” all the parameters of the scheme together with the secret.

**Quantum participants** hold their quantum shares. Each quantum participant owns one quantum share. Their task is to detect eavesdropping. This guarantees unconditional security of the scheme.

**Classical participants** hold their classical shares. There are 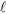 classical participants. They are responsible for secret reconstruction. Each classical participant receives their share from the dealer via a classical secure channel. Unlike in CSS, in our hybrid QSS, any *q* (*q* ≥ *m* + 1) classical participants can recover the key (secret) using their classical shares (after eavesdropping detection).

**Adversaries** includes the outsider and at most *m* insiders. The former has no valid share, while the latter is actually a legal participant with a valid share.

Finally, the proposed hybrid 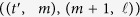 threshold QSS is defined as follows:

**Definition 1 (Hybrid** 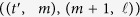 **threshold QSS).** There are *m* (where 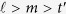) quantum participants and 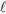 classical participants. The secret can be recovered by *m* + 1 classical participants and *t*′ quantum participants. *t*′ quantum shares from *t*′ quantum participants can be used for eavesdropping detection while *m* + 1 classical shares owned by *m* + 1 classical participants can be used to recover the secret.

**Figure 2:**
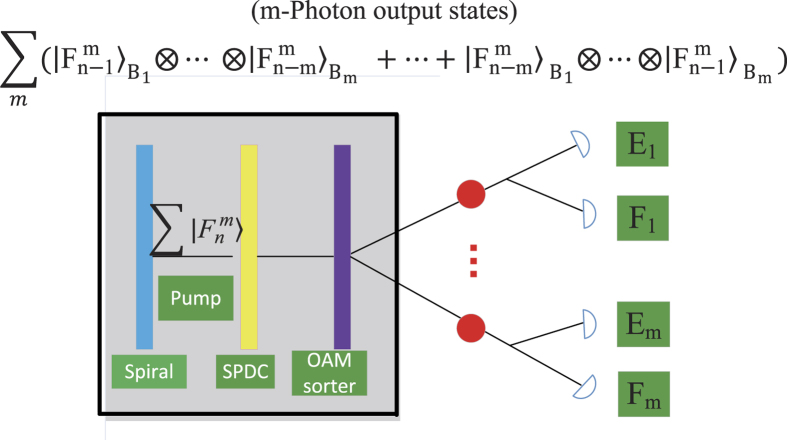
Setup for entangled states with *m*-bonacci-valued OAM on the Vogel spiral adapted from that of Simon *et al*.^31^,^32^. After entering SPDC, the entangled states are broken into *m* entangled photons. Each red circle dot represents an OAM shifter that takes the OAM in that branch to zero. Each place where lines split or cross is implied to have a BS. The portion shown has OAM values from 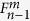 to 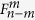 coming out of the OAM sorter. A pair of detectors *E*_*i*_ and *F*_*i*_ (*i* ∈ {1, 2, ···, *m*}) are used at the output ports of the final nonpolarizing BSs. The *E*_*i*_ sorter is used for allowing photons to arrive at the arrays of single-photon detectors when they are *m*-bonacci values, and the *F*_*i*_ sorter is used for allowing “diagonal” superposition and filtering out any non-*m*-bonacci values.

The steps of our scheme are described in details as follows:

**Dealer phase**. (1) Dealer first prepares entangled states using the *m*-bonacci number source as shown in Fig. 2. To be exact, the entangled states are prepared by the recurrence relation 
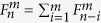
 on the Vogel spiral (refer to Simon *et al.*’s work^31^,^32^). After entering the spontaneous parametric down conversion (SPDC), the entangled states are broken into *m* entangled photons with smaller *m*-bonacci values. Each red circle dot represents an orbital angular momentum (OAM) shifter that takes the OAM in that branch to zero. Each place where lines split or cross is implied to have a beam splitter. The portion shown has OAM values from 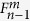 to 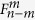 coming out of the OAM sorter.

An OAM-entangled outgoing state depends on *m*, which is as follows:


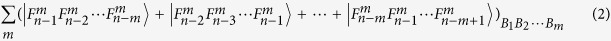


where the index *m* runs through the allowed *m*-bonacci numbers in the pump beam: 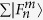. Each of the *m* quantum participants receives one entangled photon from the entangled states. In the lab of each quantum participant, there are two types of detection sorters (i.e., the *E*_*i*_ sorter and the *F*_*i*_ sorter, *i* ∈ {1, 2, ···, *m*}), directing the entangled photon to one of them at random. The *E*_*i*_ sorter is made up of an OAM sorter^33^ followed by a set of single-photon detectors. The OAM sorter transmits OAM eigenstates of various pump values into various outgoing directions, allowing them to be registered and determined in different detectors. The *F*_*i*_ sorter is used to distinguish different superpositions of the form. The states obtained in the *E*_*i*_- and *F*_*i*_- type measurements are nonorthogonal to each other. Therefore, the security of our proposed scheme is based on the fact that nonorthogonal states are indistinguishable^34^, and this principle is similar to the one used in the BB84^9^ and Ekert^10^ protocols. However, the equation (2) has an unusual feature, that is, the states detected in the *E*_*i*_-type measurement form a mutually orthogonal set among themselves, while those in the *F*_*i*_-type measurements are not all orthogonal to each other but form a chain, where each state is nonorthogonal to the two adjacent states in the chain. Moreover, for orbital angular momentum, it is not necessary for quantum states that are orthogonal in Hilbert space to associate to orthogonal vectors in the physical space. Likewise, it is not necessary for quantum states that are nonorthogonal in Hilbert space to associate to nonorthogonal vectors in the physical space. That is the second fact for our scheme’s security.

(2) The beam splitter in the quantum participant’s laboratory, sends the entangled photon to either the sorter *E*_*i*_ or the sorter *F*_*i*_ at random, where *i* ∈ {1, 2, ···, *m*}. Quantum participants *B*_1_, *B*_2_, ···, *B*_*m*_ record the sorter to which the photon goes and the detected OAM value.

(3) Dealer allocates 
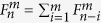
 to classical participants 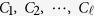 in the following way. Note that if there is something wrong with quantum channel transmission or the composition of classical participants changes (i.e., a new participant wants to join or an existing participant wishes to leave), then the dealer chooses adaptable *m*-bonacci numbers to produce new secret shares in terms of the mentioned flow of participants. This is a novel feature of our threshold adaptable secret sharing scheme.

Next the dealer uses the following algorithm to encode the secret:

**Algorithm**

1. Choose a prime *p*, 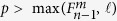.
2. Randomly and uniformly generate a number 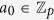 and create a polynomial: 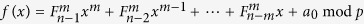, where 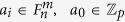.
3. Sample *f* (*x*) at 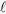 points such that *A*_1_ = *f*(1), *A*_2_ = *f* (2), …, 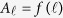. The final 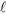 shares are (*i*′, *A*_*i*′_), for 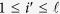.

Finally, the dealer communicates 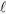 shares (*i*′, *A*_*i*′_) to appropriate classical participants, where 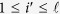.

### Eavesdropping detection phase

During the process of secret share distribution, when there is a mismatch in the entangled state photons, and if the error rate *P*_*e*_ is larger than the preset threshold *t* between Dealer and participants, they abort this communication and return to the Dealer’s phase. Otherwise, the communication continues to obtain a secure key for encrypting the shared secret, until the dealer sends an error notification or stops sending secret shares. The details are given below.

**Figure 3:**
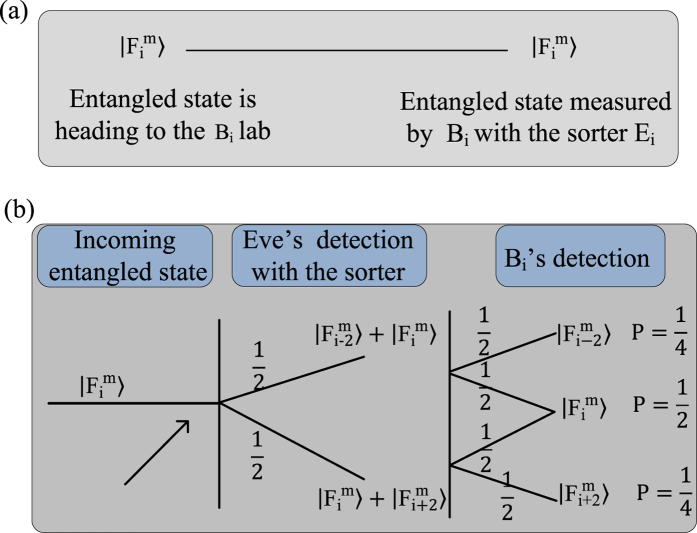
When *m* = 2, the outcome probabilities for eigenstates, where *P* denotes the probability. (**a**) When the entangled photons goes to the *E*_*i*_ sorter in *B*_*i*_’s laboratory, and Eve also happens to choose the *E*_*i*_ sorter, an incoming eigenstate should be unchanged. (**b**) When Eve chooses the *F*_*i*_ sorter, each eigenstates can turn in two different superposition detections. If one of these superpositions is transmitted to *B*_*i*_, the net outcomes are now three eigenstates that he could detect.

**Figure 4:**
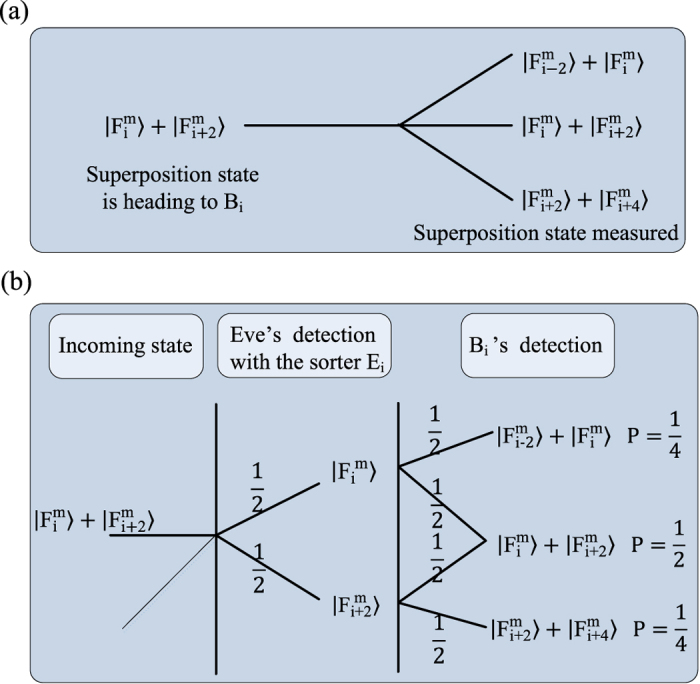
When *m* = 2, the outcome probabilities for superposition states where *P* denotes the probability. (**a**) When the entangled photons goes to the *F*_*i*_ sorter in *B*_*i*_’s laboratory without being eavesdropped, *B*_*i*_ can measure the superposition state correctly, or either of the other two superpositions states. (**b**) When Eve chooses the *E*_*i*_ sorter, she can detect two possible eigenstates with the probability of 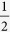 respectively, which can result in two different superpositions with the probability of 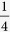 besides the original superposition state.

1. Any *m* + 1 classical participants use their shares 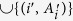 to reconstruct the polynomial 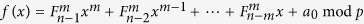. Then they can obtain the coefficients of the polynomial, i.e., 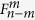, ···, 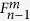. 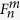 can be computed as 
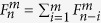
.
2. Any 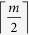 (when *m* > 2) of *B*_1_, *B*_2_, ···, *B*_*m*_ quantum participants can detect eavesdropping, by comparing the detected values with the values recovered by the *m* + 1 classical participants. We take *m* = 2 for example to illustrate how eavesdropping is detected. Suppose that an adversary Eve is eavesdropping on the quantum channel between *B*_1_, *B*_2_. Clearly, she does not know which type of a detection measurement (the sorter *E*_*i*_ or the sorter *F*_*i*_) took place in *B*_*i*_’s laboratory (1 ≤ *i* ≤ *m*). So, Eve has to guess. If the entangled photon goes to the *E*_*i*_ sorter in Eve’s laboratory when going to the *F*_*i*_ sorter in *B*_*i*_’s laboratory, or the entangled photon goes to the *F*_*i*_ sorter in Eve’s laboratory when going to the *E*_*i*_ sorter in *B*_*i*_’s laboratory, then the *B*_*i*_ measurement is going to be erroneous with the probability of 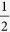. Eve’s activity is going to be detected by *B*_1_, *B*_2_, ···, *B*_*m*_ when they compare their scheme transcripts. More precisely, we have the following two cases to consider:
  1. Eve makes an *E*_*i*_-type measurement on a photon, which is actually in the eigenstate 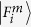. Then she will detect one of the two 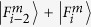 or 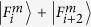, with the probability of 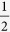, respectively. She can send a copy of it to *B*_*i*_. If *B*_*i*_ receives one of these superpositions and makes an *F*_*i*_-type measurement, she will read out one of the values 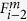, 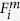, or 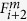, with the respective probabilities of 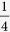, 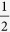, 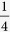 (see (a) and (b) in Fig. 3). However, she should obtain 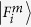 with the probability of 1 if there is no eavesdropper.
  2. Eve makes an *F*_*i*_-type measurement on a photon, which is actually in the superposition state 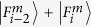. She will detect one of the two eigenstates 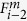, 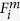, with the probability of 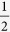, respectively. Eve may send a copy of it to *B*_*i*_. If *B*_*i*_ receives one of these eigenstates and makes an *E*_*i*_-type measurement, she will obtain one of the superpositions 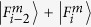 or 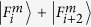 or 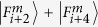, with the respective probabilities of 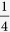, 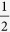, 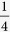 (see (a) and (b) in Fig. 4). However, she should only obtain 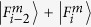 with the guaranteed probability of 1 if there is no eavesdropper.

In both cases, if Eve eavesdrops with a fraction *η* (fraction of times Eve interferes) of the trials, the fraction of times Eve guesses wrong basis is 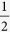, and the fraction of times wrong basis leads to error is 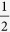. So, when quantum participant *B*_1_ compares her results with *B*_2_’s, they will find that their outcomes are inconsistent a fraction *f* of the time, which is


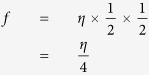


Note that in our scheme, each entangled photon binds the classical share together. Moreover, not all quantum participants are needed to detect eavesdropping when *m* > 2. This is because we can apply the recovered *m*-bonacci numbers with classical shares, to verify its consistency with the *m*-bonacci numbers carried by entangled states. Furthermore, with the detected values from the entangled photons, we can assess the security of the quantum channel (whether it is free from the adversarial activity or not).

### Secret reconstruction phase

If the participants detect eavesdropping and the error rate is higher than the preset threshold value *t*, they abort the communication and postpone the secret reconstruction phase. Otherwise, any *m* + 1 classical participants can use their classical shares to recover values 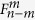, ···, 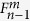, Furthermore, 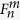 can be obtained for encoding each sub-key, which constitutes the key for encrypting the secret. With the key, the *m* + 1 classical participants can recover and further share the secret.

In this scheme, we use quantum coding to generate the key for encrypting messages. Moreover, various *m*-bonacci sequences can be used to encode the final key. That is, *m* is changeable for the key, which can address the problems of restricted quantum sources in certain settings and the membership change of participants, such as the joining of a new participant or departure of existing participants. Therefore, our scheme is more practical compared the other quantum secret sharing schemes.

## Security Analysis

First, using the technique of Simon *et al.*^31^,^32^,^35^, we show that our scheme is secure against insider and outsider attacks (Theorems 1 and 2). Later, we analyze the security of the proposed scheme and show that it is immune against a number of attacks including cloning, impersonation, replay and man-in-the-middle attacks.

**Theorem 1 (Insiders attacks).** Given the hybrid 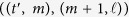 threshold QSS, and the set of classical shares available for any *k* < *m* + 1 insiders (classical participants) is 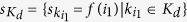, 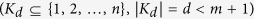, then scheme is asymptotically perfect with respect to the set of probability distributions *P*(⋅) on the secret space *S*. That is, for any 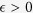, there exists an integer *p*_0_ such that for any *p* > *p*_0_ with 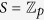, we have


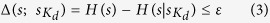


where *H*(*s*) is the entropy of *s*, the conditional entropy 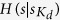 denotes the entropy of *s* conditioned on 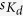, and 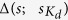 denotes the entropy loss of *s* generated by the knowledge of 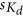.

**Proof.** It is worth noting that our proposed hybrid 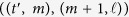 threshold QSS uses the polynomial 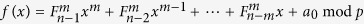, where 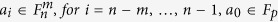. The sub-secret 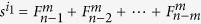. Assume that there are *d* (*d* = |*K*_*d*_| < *m* + 1) insiders. 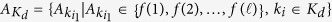 with *d* classical shares 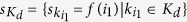, where *i*_1_ is the public information of 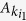, 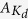 can conspire to compute


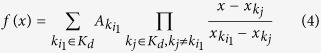


Next, we only need to consider the case of *d* = *m*, i.e., the upper bound of 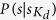, the probability of the secret with the knowledge of 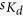. If the *s*^*i*′^ obtained by the recovered Lagrange polynomial is less than *p*, then we can have 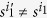, so, 
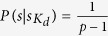
 because the recovered value 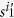 can be removed from the secret space 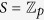. Note that *a*_0_ is chosen at random in *F*_*p*_ and thus 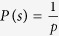. So, we have


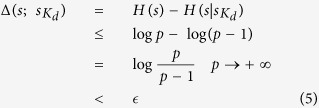


That is to say, when *p* → +∞, 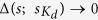. Hence, for any *k* < *m* + 1 classical participants, our proposed hybrid 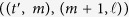 threshold QSS is asymptotically perfect with respect to the set of probability distributions *P*(⋅) on the secret space *S*. □

**Theorem 2 (Outsider attacks).** Given the hybrid 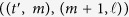 threshold QSS, and the set of the quantum participants’ shares is *Q* with |*Q*| = *m*, and an outsider has already known any subset 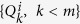 of 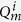, then scheme is asymptotically perfect with respect to the set of probability distributions *P*(⋅) on the secret space *S*. That is, for any 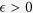, there exists an integer *p*_0_ such that for any *p* > *p*_0_ with 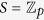, we have


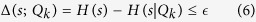


where *H*(*s*) is the entropy of *s*, the conditional entropy *H*(*s*|*Q*_*k*_) denotes the entropy of *s* conditioned on *Q*_*k*_, and Δ(*s*; *Q*_*k*_) denotes the entropy loss of *s* generated by the knowledge of *Q*_*k*_.

**Proof.** First, there are *m* + 1 (*m* > *t*′) classical participants {*C*_1_, *C*_2_, ···, *C*_*m*+1_} and *t*′ quantum participants {*B*_1_, *B*_2_, ···, *B*_*t*′_}. Suppose that an outsider has already known 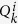 quantum shares and recovered the value 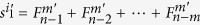. Let us examine the probability of 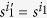, and 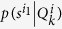 is the probability of the sub-secret 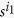 with the knowledge of 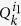. 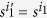 means 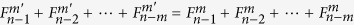.

On the one hand, for the party who detects a particular *m*-bonacci number, there is still an *m*-fold uncertainty about the *m*-bonacci number other parties detect in ref. 32. For example, when 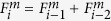, if the outsider detects the value 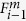, the other party’s value can be 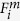 or 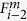. Moreover, due to the quantum no-cloning theorem^13^, outsiders will be detected when eavesdropping over the quantum channels (see Figs 3 and 4). Therefore, 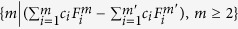 are uniformly distributed. To some degree, this suggests the sub-secret 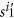 and 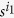, even *s*′ and *s* are actually independent of each other.

On the other hand, the quantum participants’ shares are independent of the classical participants’ shares. Moreover, the quantum participants’ shares are used for eavesdropping detection rather than key generation. Consequently, outsiders cannot use their quantum shares directly as they do in Cleve *et al.*’s QSS^14^ to obtain the secret. Therefore, outsiders cannot have a better way to get the secret except to assume *s*′ = *s*.

Second, as we know, the classical shares are allocated in Shamir’s SS^1^, which are uniformly distributed over 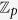. Given that 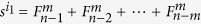, and 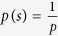. The probability for outsiders to successfully obtain the values without being detected is 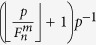, where 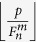 means that the largest integer is less than or equal to 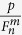. Hence, the entropy loss of the secret satisfies


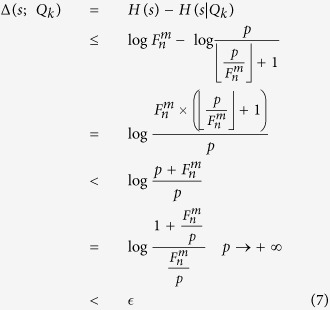


That is to say, when *p* → +∞, Δ(*s*; *Q*_*k*_) → 0. Hence, our proposed hybrid 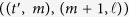 threshold QSS is asymptotically perfect with respect to the secret space *S*. Here, 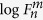 is the abbreviation of 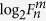. □

### Resistance against cloning and impersonation attacks

Our scheme is based on the fact that the nonorthogonal states are indistinguishable, so it is immune against cloning and impersonation attacks. Even if Eve successfully detects the value of an *m*-bonacci-value-entangled photon, according to the quantum no-cloning theorem, she is unable to clone any other undetected *m*-bonacci-value-entangled photon.

Let us consider impersonation attacks. A possible strategy Eve can use is to capture the original *m*-bonacci-value-entangled photon and send a fake *m*-bonacci-value-entangled photon, say 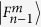, to other quantum participants. To be exact, after Dealer sends the state, Eve simply captures the original state and stores it. She then sends one *m*-bonacci-value-entangled photon from the fake state 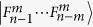 to other quantum participant *B*_*i*_. However, due to the particular encoding used in our scheme, the *m*-bonacci-value-entangled photon varies and the impersonation attack is easy to be detected. For example, if she detects that the value of entangled photon is 2 from 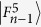, she sends one 3-bonacci-value-entangled photon as the fake state. Obviously, the impersonation attack does not succeed.

### Resistance against replay attacks

Our scheme is immune against replay attacks. A replay attack is such an attack that a valid data transmission is maliciously or fraudulently repeated or delayed. Because we use adaptable *m*-bonacci sequences for preparing *m*-bonacci-sequence entangled states, the quantum and classical shares change accordingly. Moreover, due to the use of varying *m*-bonacci numbers to prepare entangled states, Eve cannot know which *m*-bonacci sequence is really used every time. For example, suppose that for the fourth subkey, 3-bonacci sequences are used, however, 6-bonacci sequences are used for the fifth subkey. As a result, it is impossible to launch an impersonation attack by inserting the used *m*-bonacci sequences for the subkey. Therefore, our scheme is immune to replay attacks.

### Resistance against man-in-the-middle attacks

A man-in-the-middle attack is an attack, in which Eve intercepts the transmitted entangled photons and replays other entangled photons. We now show that our scheme provides resistance against the man-in-the-middle attacks. First, quantum channels are authenticated; second, for the party who detects a particular *m*-bonacci number, there is still a *m*-fold uncertainty about the *m*-bonacci number other parties detect. Suppose that the eavesdropper Eve, is in possession of an entangled state analyzer for the *m*-bonacci-value entangled states. If so, she will be able to distinguish 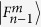, 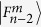, ···, or 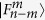 when Dealer sends one of them. However, in the detected sorter, she has a random outcome. Suppose the eavesdropping strategy is to resend the *m*-bonacci-value entangled state according to the result of her *m*-bonacci-value entangled state analysis. In order to detect the eavesdropper, we should consider what happens if, for instance, the state 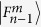 is sent by Dealer. In one 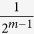
th of the cases Eve chooses the right sorter, and resends the *m*-bonacci-value entangled state perfectly. In the remaining 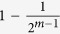 of the cases Eve chooses the wrong sorter and sends the superposition state 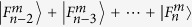. These states will be correctly detected by *B*_1_, *B*_2_, ··· *B*_*m*_ together with any *m* + 1 classical participants. By adding all the probability of causing an error becomes 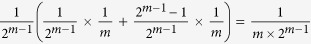, which is the same as two-state cryptography. In a manner similar to the two-state cryptography, it is also possible to launch a more complex eavesdropping attack using an ancilla, or measuring in an intermediate basis compared to the {0, 1} bases. However, the eavesdropper is still detectable, and the fundamental security remains. Hence, our scheme provides resistance against the man-in-the-middle attacks.

## Discussion

Simon *et al.*^31^ used positive and negative OAM pumps to improve information capacity which can only be doubled, and their scheme needs a joint quantum operation. Moreover, with 2-bonacci values used alone, to multiple the information capacity, larger 2-bonacci values should be used, and the available bandwidth becomes more of a challenge. Also, they argued that though lower error rates can be achieved by the use of higher-2-bonacci values (Fibonacci numbers), the transmission distances are shorter. While the longer distances can be achieved by the use of lower-2-bonacci values, the error rates are higher. When only 2-bonacci values are used, it is hard to satisfy the requirements of lower error rates and longer transmission distances. Based on these mentioned problems, we incorporate *m*-bonacci sequences into both quantum and classical coding, with reverse Huffman-Fibonacci-tree coding, to achieve higher-capacity and lower-bandwidth hybrid threshold adaptable QSS scheme.

### The information capacity

Given the above conclusion, proper *m*-bonacci values can be chosen to achieve the lower error rates and longer distances. Hence, we propose to use Huffman coding tree to encode *m*-bonacci numbers with the greedy algorithm, which can greatly improve the coding capacity, thus reducing the use of entangled photons. To be exact, for fixed *m*-bonacci number sets, we use binary representations of *m*-bonacci numbers based on Fibonacci numbers of order *m* ≥ 2 (see Equation (11)). Hence, this paper extends Simon *et al.*’s quantum key distribution protocol presenting a novel feature, where Fibonacci numbers 1, 2, 3, 5, 8, 13, 31, 34 are used. According to the method of reverse Huffman-Fibonacci-tree coding in Eqs (11) and (12) of the following section, each *m*-bonacci number can then represent a binary string as follows:


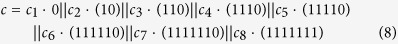


where 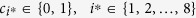, and 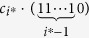 denotes when i^*^ = 1, the corresponding codes are 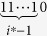; when i^*^ = 0, there are no corresponding codes.

Therefore, the information capacity of each *m*-bonacci number is


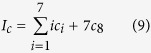


Take 4 = 3 + 1, 7 = 5 + 2, 15 = 13 + 2, 16 = 13 + 3, 24 = 21 + 3, 29 = 21 + 8, 31 = 21 + 8 + 2, 32 = 21 + 8 + 3 for example (as shown in Table 1, according to Equations (11) and (12), their binary Fibonacci representations would be as follows:


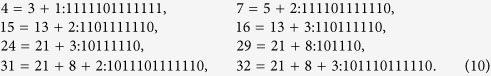


It can be seen from Equation (10), compared with the high-capacity coding in terms with Simon *et al.*’s protocol, in which they double the information capacity per photon, we multiply the information capacity. In the above example, the average bits per photon is 10.375. If the size of the key is 360,000, we need to prepare about 36,000 rather than 90,000 entangled states, making our scheme more practical. This is because it is difficult and costly to prepare entangled states.

Besides the information capacity, Table 2 compares the features of our proposed scheme with those of the secret sharing schemes in refs 1, 14, 24 and 26. The comparison suggests that our secret sharing scheme is more suitable for real-world applications. Distinct from the well-known Shamir’s classical secret sharing scheme^1^ against secret leakage, our proposed scheme can both detect eavesdropping and protect the secret from leaking. Meanwhile, compared with QSS schemes^14^,^24^,^26^, our scheme can achieve the adaptability and flexibility based on the following two facts: 1) the various *m*-bonacci values are used to adapt to participant mobility; 2) the *m*-bonacci values are encoded in Lagrange polynominal, and as a result, any *t*′ quantum participants and any *m* + 1 classical participants can recover the secret. Due to the no-cloning theorem and its impact on parameters, for QSS schemes^14^,^24^,^26^,^31^, the parameters 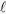 and *t*′ must satisfy the requirement of 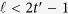, and once *t*′ quantum participants are fixed, other quantum participants are unable to participate in the recovery of the secret. However, in our scheme, the parameters 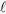 and *t*′ can be arbitrary, and our scheme is robust when a new participant joins or an existing participant leaves.

Unlike eavesdropping detection in QSS schemes^14^,^24^,^26^, due to entangled states prepared by *m*-bonacci sequences, the detection is possible by any subset that contains at least 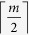 participants. That is to say, none of the number of threshold value quantum participants are required to reach a consensus in order to reveal eavesdropping. Because the method of the Huffman-Fibonacci coding is employed in our scheme, the classical bits denoted by every *m*-bonacci number are significantly improved, from four bits at most to more than ten bits using a similar experimental setup. Consequently, our scheme can greatly improve the coding capacity, thus reducing the use of entangled photons which are expensive and difficult to prepare. Moreover, to generate the secret, the *m*-bonacci sequences encoded in Lagrange interpolation polynomials and the Huffman-Fibonacci coding are applied. So, compared with the CSS in ref. 1 where the size of secret shares is the same as that of the secret itself, our scheme allows the former to be of much smaller than the secret itself. To be exact, the smaller *m*-bonacci sequences such as *m* = 2, 3, 4, 5, 6 can be used in our proposed scheme, the pump values in Fig. 1 is smaller and the size of classical shares is much smaller since the size of the prime is much smaller than that used in ref. 1. In addition, we generate secret shares for blocks generation, thus significantly reducing the bandwidth compared with ref. 1.

For the communication overhead, there are *m* quantum and 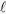 classical participants. Let *P* and *p* (*P* ≫ *p*) be primes, then the communication overhead in ref. 1 is *n*|*P*| bits, where |*P*| and |*p*| denote the bit number of *P* and *p*. It can be known that the size of the key is |*P*|, so, |*P*| photons are prepared. In ref. 14, only quantum channel is used, so, the communication overhead is *n*|*P*| qubits. The ref. 24 proposes a hybrid quantum secret sharing, in which both quantum and classical channel are used. There are 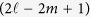 quantum participants and 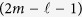 classical participants. Therefore, the quantum communication overhead for the scheme in ref. 26 is 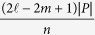 qubits, and the classical communication overhead is 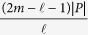 bits. In our scheme, the quantum communication overhead is at most 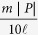 qubits, and the classical communication overhead is 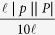 bits.

In conclusion, we combine the Huffman-Fibonacci quantum coding with Lagrange polynomials to achieve threshold adaptable QSS. The key point of our scheme is that it does not suffer from the restriction derived from the quantum no-cloning theorem, because it permits secret sharing for arbitrary values of parameters 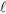 and *m* + 1 provided that 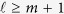. We use the Huffman coding tree to encode the obtained *m*-bonacci numbers, aiming at improving the coding capacity greatly, and thus incurring a low communication overhead. When compared to the existing QSS schemes, there is an improvement in sharing the secret without joint quantum operations. Meanwhile, our scheme still works when there are dynamic changes in comparison with existing quantum secret sharing, such as the unavailability of some quantum channel, the arrival of new participants and the departure of participants.

## Methods

### Huffman-Fibonacci coding

Fraenkel and Klein^29^ showed that any integer including an *m*-bonacci number can be represented by a binary string of length *r*, *c*_*r*_, *c*_*r*−1_, …, *c*_2_, *c*_1_ such that


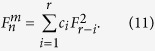


When one uses the following procedure to produce it, the Equation (11) will be unique. Given the integer 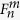, find the largest Fibonacci number 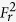 smaller or equal to 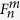; then continue recursively with 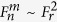. Therefore, for coding, we explore the properties of Fibonacci representations for variable-length encoding, especially the trade-off between their robustness and their scalability efficiency. To be exact, we use the Huffman coding in ref. 36 and the greedy algorithm in ref. 37 to improve the coding capacity of the detected *m*-bonacci values.

**Figure 5:**
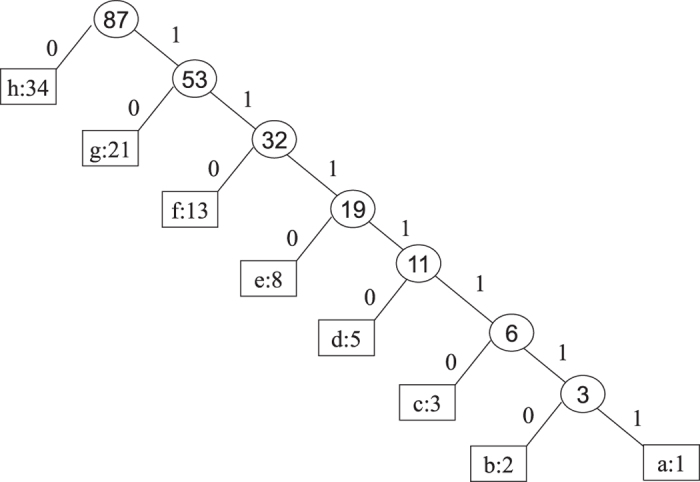
The Huffman-Fibonacci coding tree for the eight Fibonacci numbers (as symbol frequencies) from 1 to 34.

*m* + 1 classical participants encode the reconstructed *m*-bonacci value based on the fact that the higher the frequency at which Fibonacci numbers appear in *m*-bonacci values, the longer the set of binary codes, which is opposite to the idea of Huffman coding. Let the binary codes of 
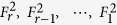
 be 
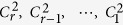
 respectively. So, the binary codes of 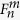 are represented by the concatenation of 
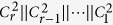
. For example, if the frequencies from 1 to 34 which appear in *m*-bonacci values decrease, the coding is as follows (see Fig. 5);


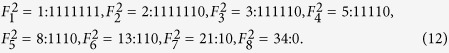


So, the available reconstructed 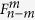 is used in terms of Eqs (9) and (12), and a sub-key can be obtained. The key can be obtained by concatenating all the sub-keys generated in the same way.


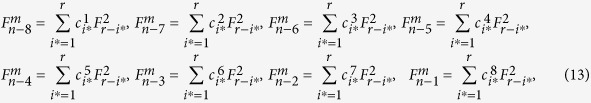


where 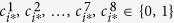.

For example, if the final detected values of available entangled states are 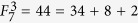, 
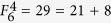
, ···, their corresponding coding is 011101111110, 101110, ··· in terms of Eq. (12). The key can be established with 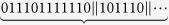 concatenated for secret sharing. In other words, any *m* + 1 classical participants can share the secret encrypted by the key using the one-time-pad encryption.

## Additional Information

**How to cite this article**: Lai, H. *et al.* Hybrid threshold adaptable quantum secret sharing scheme with reverse Huffman-Fibonacci tree coding. *Sci. Rep.* **6**, 31350; doi: 10.1038/srep31350 (2016).

## Figures and Tables

**Table 1:** Fibonacci numbers of order *m* = 2, 3, 4, 5, 6.

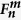	*n* = 1	*n* = 2	*n* = 3	*n* = 4	*n* = 5	*n* = 6	*n* = 7	*n* = 8	*n* = 9	*n* = 10
*m* = 2	1	2	3	5	8	13	21	34	55	89
*m* = 3	1	2	4	7	13	24	44	81	149	274
*m* = 4	1	2	4	8	15	29	56	108	208	401
*m* = 5	1	2	4	8	16	31	61	120	236	464
*m* = 6	1	2	4	8	16	32	63	125	248	492

**Table 2:** Performance comparison of HQSS with previous QSSs.

Schemes	Ref. 1	Ref. 14	Ref. 24	Ref. 26	Our scheme
Adaptable threshold	No	No	No	No	Yes
Flexible threshold	Yes	No	No	No	Yes
Robustness	High	Low	Low	Low	High
Detect eavesdropping	No	Yes	Yes	Yes	Yes
The information capacity		1bit	1bit	1bit	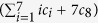 bits
Classical communication overhead	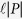 bits	0bit	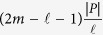 bits	0bit	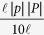 bits
Quantum communication overhead	0	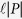 qubits	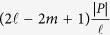 qubits	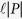 qubits	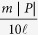 qubits
Hybrid quantum secret sharing	No	No	Yes	Yes	Yes

*m* is the threshold value, and 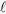 denotes 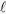 classical or quantum participants, *P* and *p* (*P* ≫ *p*) are primes which are used in ref. 1 and our scheme, and |*P*| and |*p*| denote the bit number of *P* and *p*.
